# A novel image encryption algorithm based on fractional order 5D cellular neural network and Fisher-Yates scrambling

**DOI:** 10.1371/journal.pone.0236015

**Published:** 2020-07-15

**Authors:** Xingyuan Wang, Yining Su, Chao Luo, Chunpeng Wang

**Affiliations:** 1 School of Information Science and Technology, Dalian Maritime University, Dalian, China; 2 School of Information Science and Engineering, Shandong Normal University, Jinan, China; 3 School of Information, Qilu University of Technology (Shandong Academy of Sciences), Jinan, China; Lanzhou University of Technology, CHINA

## Abstract

This paper proposes a new chaotic image encryption algorithm. Firstly, an original phased composite chaotic map is used. The comparative study shows that the map cryptographic characteristics are better than the Logistic map, and the map is used as the controller of Fisher-Yates scrambling. Secondly, with the higher complexity of the fractional-order five-dimensional cellular neural network system, it is used as a diffusion controller in the encryption process. And mix the secret key, mapping and plaintext, we can obtain the final ciphertext. Finally, the comparative experiments prove that the proposed algorithm improves the encryption efficiency, has good security performance, and can resist common attack methods.

## 1 Instruction

With the rapid development of the computer industry, more and more multimedia information needs to ensure its encryption status during transmission to prevent others from gaining privacy and conduct improper behavior. Digital images, because of their large data volume and high data correlation, combined with information image and vividness, have become one of the important means for people to express information. A good encryption algorithm for images is very necessary. Double random phase coding (DRPE) [1] technology provides a theoretical basis for many subsequent optical encryption systems, but it is vulnerable to attack [2]. Therefore many experts have proposed optical image encryption based on Fresnel transform domain [3], gyration transform domain [4], fractional Merlin transform domain [5]. For example, Zhou et al. proposed an image compression encryption algorithm based on compressed sensing and fractional Merlin transform. In order to reduce the problems caused by linear characteristics, chaos is a theoretical system sensitive to initial value conditions, just meets people's expectations of passwords in the encryption process. Especially in recent times, chaotic encryption systems are increasingly used for image encryption [6–10]. For example, Ye et al. used the Logic-tent map for visually meaningful image encryption.

Chaos is a complex nonlinear dynamic system. Chaotic phenomena are a kind of random process in nonlinear deterministic systems. Because chaotic signals have noise-like, initial value sensitivity and long-term unpredictability, they are especially suitable for secure communication. technology. Hyperchaotic systems have higher security performance because they can generate more complex dynamic behaviors, have stronger randomness and unpredictability. In recent years, chaos has been used as a research hotspot for people's attention. The color image, by changing the existing order of the image, arranges the pixels according to some operations to form a noise-like image to achieve the encryption effect [11]. So far, some color image encryption algorithms based on chaos theory have been proposed [12–15], while high-dimensional chaotic systems, especially hyperchaotic systems, have large key spaces, complex and unpredictable nonlinear behavior, using hyperchaotic systems to encrypt data will improve the security of the cryptosystem. The research finds that the chaotic characteristics exhibited by CNN make its application in secure communication more and more important. Using the chaotic characteristics of CNN to design the image encryption scheme, the advantages are as follows: Although the dynamic equation of CNN is simple in form, there are chaotic attractors in a large parameter range, and the dynamic behavior is complex; the parameters in CNN dynamic equation are more. The encryption scheme with large key space can be designed; CNN dynamic equation can directly generate a better random matrix, which can design a digital image encryption scheme more conveniently.

Therefore, this paper proposes a color image encryption algorithm based on cellular neural network. The initial key is artificially selected. After the processing of the staged chaotic map, the generated sequence is used as a random number generator to perform Fishery's scrambling, and then the scrambled image is serialized, and the fractional five-dimensional is used. The sequence obtained after the diffusion of the cellular neural network is subjected to secondary diffusion, and finally the ciphertext is obtained. Finally, through simulation experiments, it can be seen that this algorithm has improved security compared with previous algorithms, and is suitable as a way of image encryption.

## 2 Introduction to chaotic systems

### 2.1 Fractional 5-dimensional cellular neural network model

The cellular neural network was proposed by Chua and Yang in a combination of cellular automata and Hopfield neural networks. The cellular automatic machine composed of a large number of basic units provides a good model basis for a complex self-organizing structure. The Hopfield neural network constructed by the storage system and the binary neural network can realize the extreme convergence of the system by recursively. They can achieve weight controllable, dual-transmission and local connectivity through improved system networks. Due to its excellent characteristics, cellular neural networks are widely used in prediction, pattern recognition, and control. However, there is not much literature on applying the hyperchaotic characteristics of CNN to image encryption. In many literatures, the chaotic sequences generated by CNN are used for encryption, but due to the defects of its own algorithm, its encryption effect cannot meet the resistance of existing attack methods. Therefore, this paper will use the use of fractional-order 5D neural network to make it better applied to the field of image encryption against existing attacks.

According to its schematic diagram, each neuron cell in CNN can be expressed by Eq (1).

$$\frac{dx_{ij}(t)}{dt}=-\frac{1}{R}x_{ij}(t)+\sum_{C(k,l)\in N_{r}(i,j)}A(i,j;k,l)+\sum_{C(k,l)\in N_{r}(i,j)}B(i,j;k,l)u_{kl}(t)+I,$$ (1)

where *x*_*ij*_(*t*) is the state variable of the cell (*i*,*j*); *I* indicates the external output of the network; *u*_*kl*_(*t*) indicates the corresponding input voltage of the cell (*i*,*j*); *y*_*ij*_(*t*) is the corresponding output of the cell (*i*,*j*), Its output function *f*(*x*_*ij*_) is a piecewise linear function, whose expression is shown in Eq (2):

$$f(x_{ij})=\frac{1}{2}(|x_{ij}+1|-|x_{ij}-1|),$$ (2)

In the research, we simplified the state equation of CNN for the convenience of research. This paper introduces a simplified version of the CNN model and divides its fractional order into five dimensions, in the form of Eq (3).

$$\frac{d^{q_{i}}x_{i}}{d^{q_{i}}t}=-x_{j}+a_{j}p_{i}+\sum_{\begin{array}{l} k=1\\ k\neq j \end{array}}^{5}a_{jk}p_{k}+\sum_{k=1}^{5}s_{jk}x_{k}+I_{j},$$ (3)

The CNN parameters of the 5 cells are as follows:

$$a_{1}=a_{2}=a_{3}=a_{5}=0,a_{4}=202,a_{jk}=0(j,k=1,2,3,4,5,j\neq k),I_{j}=0(j=1,2,3,4,5)$$

$$S_{jk}=\left[\begin{array}{ccccc} 1 & 0 & -1 & -1 & -1\\ 0 & 3 & 1 & 0 & 0\\ 11 & -12 & 1 & 0 & 0\\ 92 & 0 & 0 & -94 & -1\\ 0 & 0 & 15 & 0 & -1 \end{array}\right].$$ (4)

Then, Eq (3) can be

$$\left\{ \begin{array}{l} \frac{d^{q_{1}}x_{1}}{dt^{q_{1}}}=-x_{1}+S_{11}x_{1}+S_{13}x_{3}+S_{14}x_{4}\\ \frac{d^{q_{2}}x_{2}}{dt^{q_{2}}}=-x_{2}+S_{22}x_{2}+S_{23}x_{3}\\ \frac{d^{q_{3}}x_{3}}{dt^{q_{3}}}=-x_{3}+S_{31}x_{1}+S_{32}x_{2}+S_{33}x_{3}\\ \frac{d^{q_{4}}x_{4}}{dt^{q_{4}}}=-x_{4}+S_{41}x_{1}+S_{44}x_{4}+S_{45}x_{5}\\ \frac{d^{q_{5}}x_{5}}{dt^{q_{5}}}=-x_{5}+S_{53}x_{3}+S_{55}x_{5} \end{array}\right..$$ (5)

**Fig 1:**
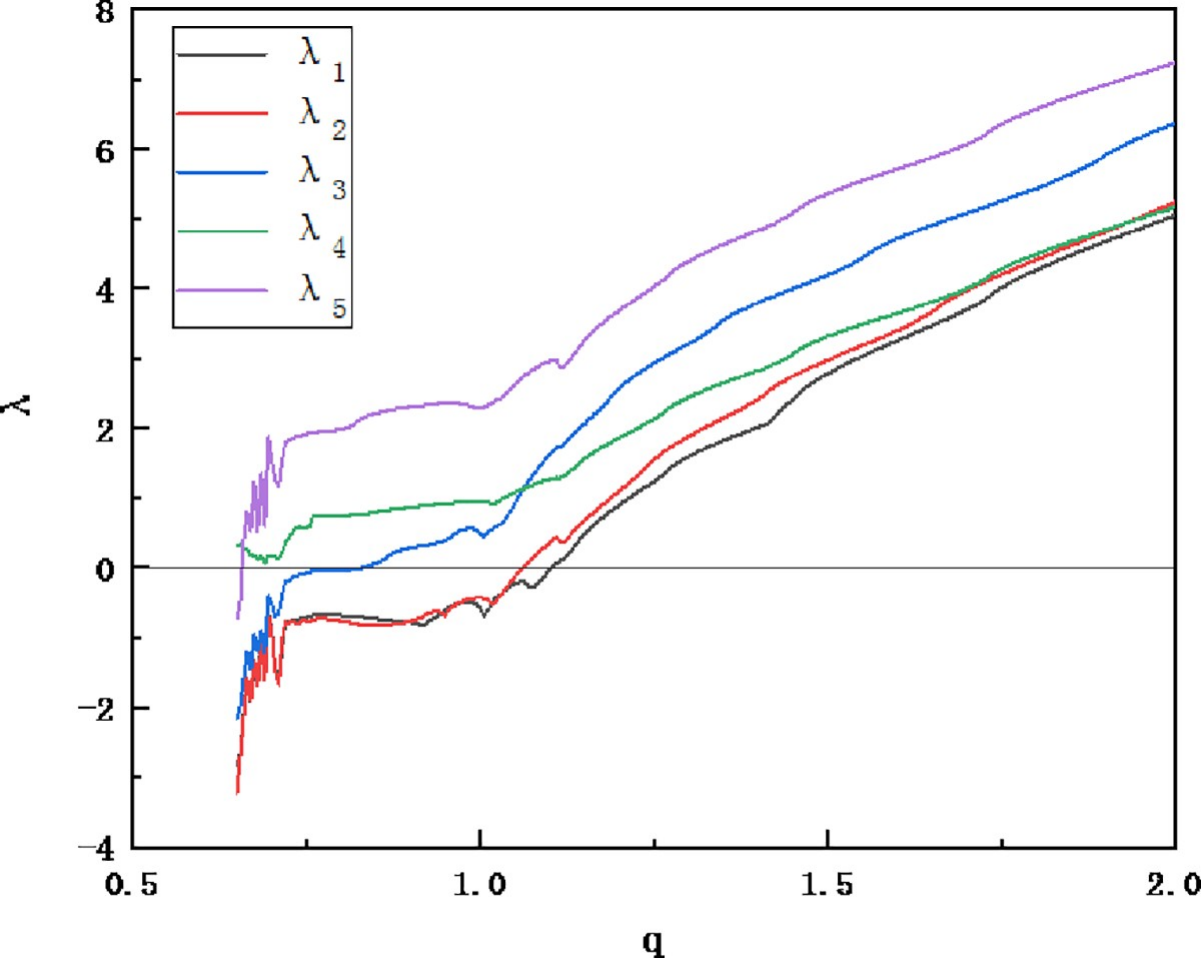
Fractional-order cellular neural network Lyapunov exponential map.

**Fig 2:**
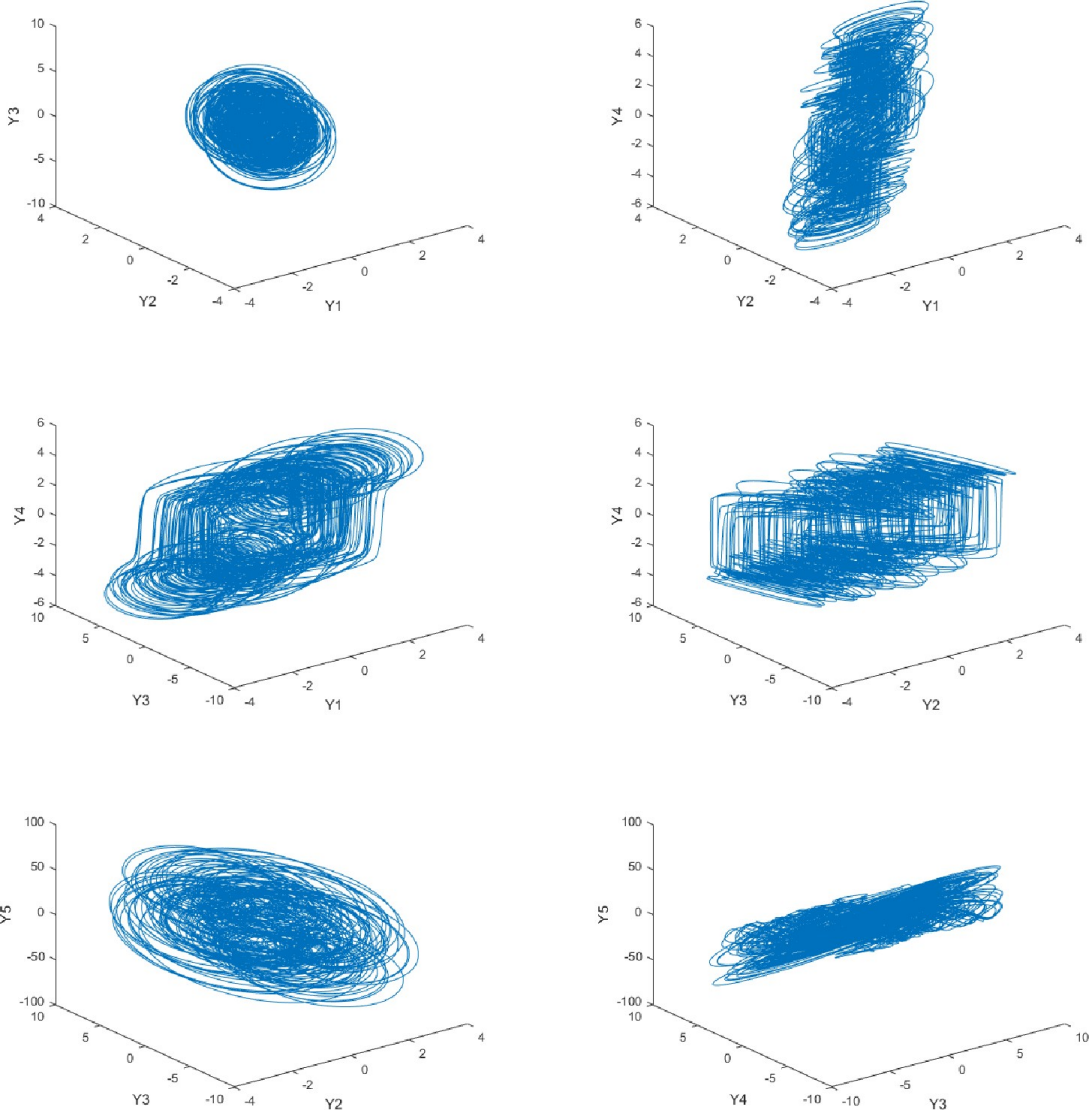
3-D projection of chaotic attractors for fractional-order cellular neural networks.

Solve Eq (5) using the Runge-Kutta method with a step size of 0.005, set *x*_1_ = 0.1,*x*_2_ = *x*_3_ = *x*_4_ = *x*_5_ = 0.2, its Lyapunov exponent diagram is shown in Fig 1. It can be seen that, when *q*_1_ = *q*_2_ = *q*_3_ = *q*_4_ = *q*_5_ = 0.98, The Lyapunov index is -0.49121, -0.45898, 0.55644, 0.94533, 2.33257. Therefore, it can be considered that the system is already in a chaotic state at this time, and its chaotic attractor map is shown in Fig 2.

### 2.2 Staged composite chaotic mapping model

Although one-dimensional chaos can generate pseudo-random sequences, it is often used in image encryption and neural networks [16–18], but due to its low complexity, it is easy to be predicted, which reduces the security of the entire encryption system. We use a phased compound chaotic map composed of two one-dimensional chaotic maps (Logistic map and Tent map) [19]. The specific operations are as follows:

The Logistic map is divided into two parts and its form is

$$x_{n+1}=\left\{ \begin{array}{l} 4\mu x_{n}(0.5-x_{n}),0\leq x<0.5\\ 4\mu x_{n}(1-x_{n})(x_{n}-0.5),0.5\leq x\leq 1 \end{array}\right.,$$ (6)

**Fig 3:**
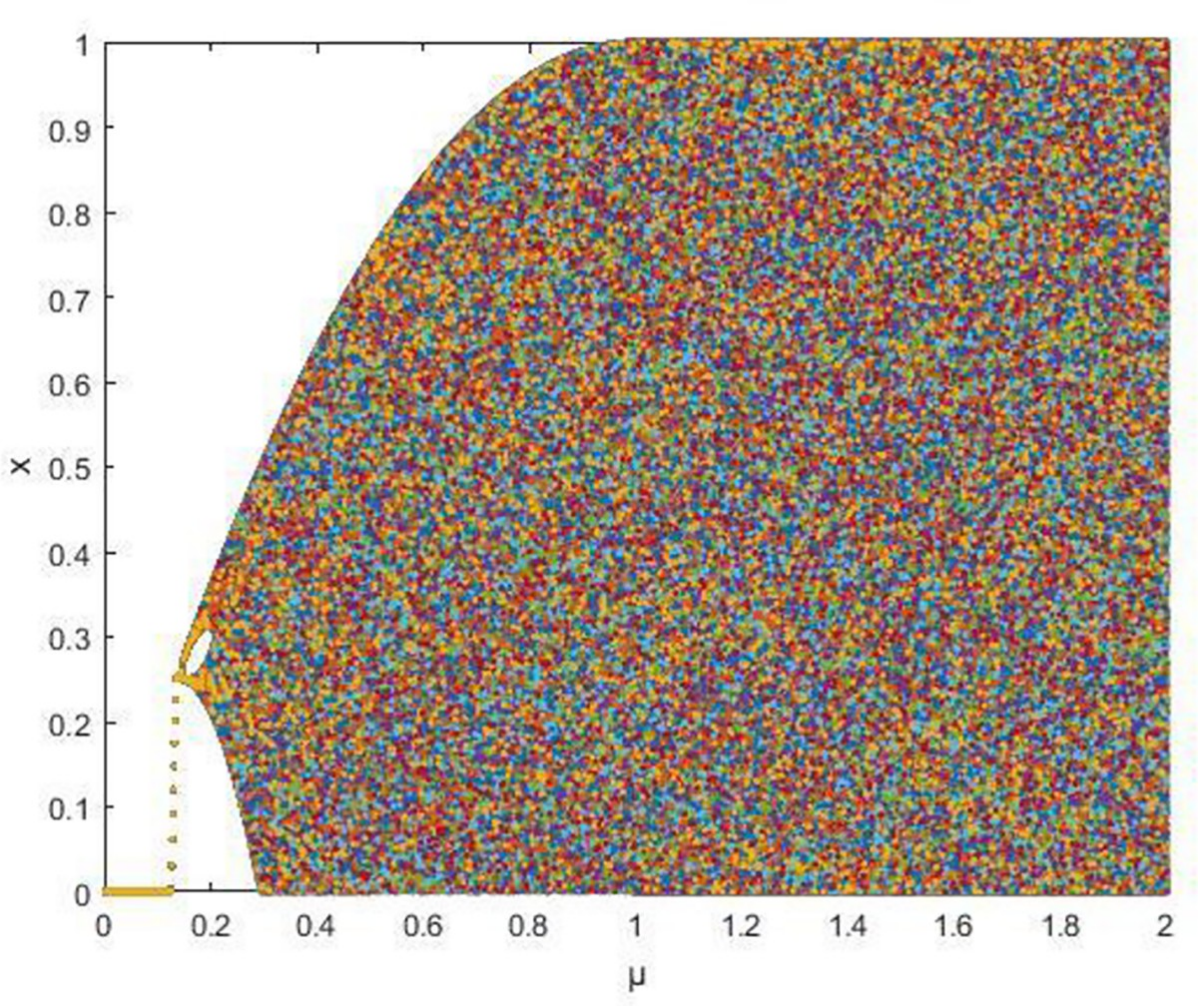
Bifurcation diagram of staged composite chaotic map.

Divide the Tent map into four parts, the phased compound chaotic map can be obtained by taking the Eq (4)

$$x_{n+1}=\left\{ \begin{array}{l} 16\mu x_{n}(0.5-\mu x_{n}),0\leq x<0.25\\ 16\mu (0.5-x_{n})(0.5-\mu (0.5-x_{n})),0.25\leq x<0.5\\ 16\mu (x_{n}-0.5)(0.5-\mu (x_{n}-0.5)),0.5\leq x<0.75\\ 16\mu (1-x_{n})(0.5-\mu (1-x_{n})),0.75\leq x\leq 1 \end{array}\right.,$$ (7)

where *μ*∈[0,2], *x*_*i*_∈[0,1], its bifurcation diagram is shown in Fig 3, the map has entered a chaotic state at *μ*>0.33.

## 3 Algorithm description

### 3.1 Scrambling process based on phased composite chaotic map

Fisher-Yates scrambling is generally a random arrangement that produces a finite set. The Fisher-Yates random scrambling algorithm is unbiased, so each permutation is equally possible. The currently used Fisher-Yates random scrambling algorithm is quite effective, and the time required is proportional to the number to be randomly scrambled. The amount of storage space required is required.

Fisher-Yates scrambling is mainly based on a certain rule, starting from the last element of a sequence, and exchanging numbers with a previous position. If the rules for selecting numbers are random, the entire scrambling process is also random. However, the random process does not make the scrambling process reversible, so the use of Fisher-Yates scrambling in this paper is based on the pseudo-randomness of the phased composite chaotic map mentioned above. Because of its good pseudo-randomness, it can undertake the task of random scrambling required, and can complete the restoration of the image with the key.

### 3.2 Diffusion based on fractional-order 5-dimensional cellular neural networks

In the diffusion phase, this paper uses a fractional-order 5-dimensional cellular neural network as a diffusion method to spread the pixel values of the scrambled image to ensure that the plaintext information can be diffused into each pixel to make it against common attacks. Can have good resistance. In this paper, the diffusion process mainly selects the sequence of the corresponding cellular neural network according to the key, and XORs the ciphertext and the plaintext, and then generates a new ciphertext. The specific process will be described in detail below.

### 3.3 Encryption steps

We use a grayscale image of size *M*×*N*, where *M* is the width of the image and *N* is the height of the image. This article uses Matlab to process digital images, so the default is 1 as the beginning of the array instead of 0.

In the scrambling process, *x*,*μ* is input as a key, and then the key is processed according to Eq (7) to obtain a required phased chaotic map sequence *L*. Then use Fisher-Yates to scramble, use the first scramble, and then scramble to the whole picture. The scrambling process is to first extract the required scrambling rules from the phased composite map, as shown in Eq (8).

$$L^{\prime }=L(1+(j-1)\times N:j\times N),$$ (8)

where *j* is the encrypted column, and after the scrambling rule is obtained, the column *j* is scrambled, and the scrambling method is as shown in Eqs (9)–(12).

$$L_{Final}=floor(L^{\prime }\times (N-k+1))+1,$$ (9)

$$temp=Image_{j}(L_{Final}(N-k+1),j),$$ (10)

$$Image_{j}(L_{Final}(N-k+1),j)=Image_{j}(N-k+1,j),$$ (11)

$$Image_{j}(N-k+1,j)=temp,$$ (12)

where Eq (9) is performed by converting *L*′ into integers between [1,*N*], and Eqs (10)–(12) accomplish the scrambling of the column *j*, *k* represents the number of elements that have been scrambled in column *j*. Repeat this process until each column is scrambled and then finished. After the column is scrambled, the row elements are scrambled with the same rules. At this point, the entire scrambling process is completed.

After the scrambling is completed, a semi-ciphertext *Image*_*semi*_ is obtained, and then the semi-ciphertext is diffused. First use the initial key to make it integer, as shown in Eq (13)

$$key=\mathrm{mod}(L(1)\times 10^{14},256),$$ (13)

Then perform the reconstruction as shown in Eq (14) on the semi-ciphertext *Image*_*semi*_,

$$Image_{semi}^{\prime }=reshape(Image_{semi},M\times N,1),$$ (14)

after that, the chained conduction diffusion is performed on the reconstructed $Image_{semi}^{\prime }$, as shown by Eqs (15) and (16).

$$\left\{ \begin{array}{l} C_{1}=(Image_{semi}^{\prime }(1)⊕key)⊕(\mathrm{mod}(Y(1,\mathrm{mod}(key,5)+1)\times 10^{5},256))\\ C_{i}=(Image_{semi}^{\prime }(i)⊕C_{i-1})⊕(\mathrm{mod}(|Y(i,\mathrm{mod}(C_{i-1},5)+1)|\times 10^{5},256))(i\neq 1) \end{array}\right.,$$ (15)

**Fig 4:**
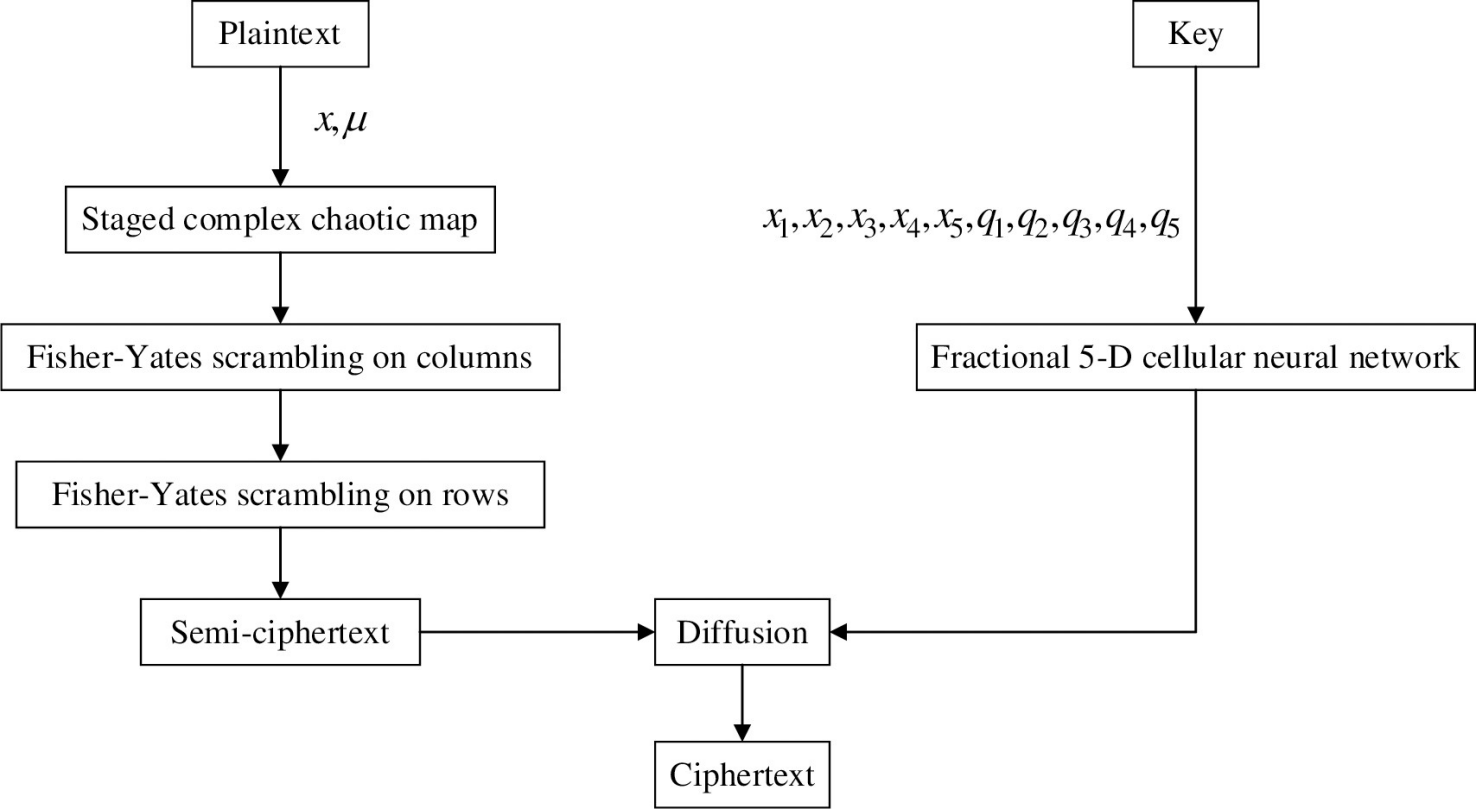
Encryption flow chart.

C=reshape(C,M,N), (16)

where *Y* is the sequence of the generated fractional-order cellular neural network. There are five groups, and the required parameters are *x*_1_,*x*_2_,*x*_3_,*x*_4_,*x*_5_,*q*_1_,*q*_2_,*q*_3_,*q*_4_,*q*_5_, *C* is ciphertext. At this point, the encryption process ends, and the process of the encryption process in this paper is shown in Fig 4.

### 3.4 Image decryption

In the decryption process of this paper, in the case of known keys and ciphertext, the required parameters, such as *x*,*μ* and *x*_1_,*x*_2_,*x*_3_,*x*_4_,*x*_5_,*q*_1_,*q*_2_,*q*_3_,*q*_4_,*q*_5_, can be generated to solve the original image.

## 4 Experimental results and analysis

### 4.1 Experimental result

**Fig 5:**
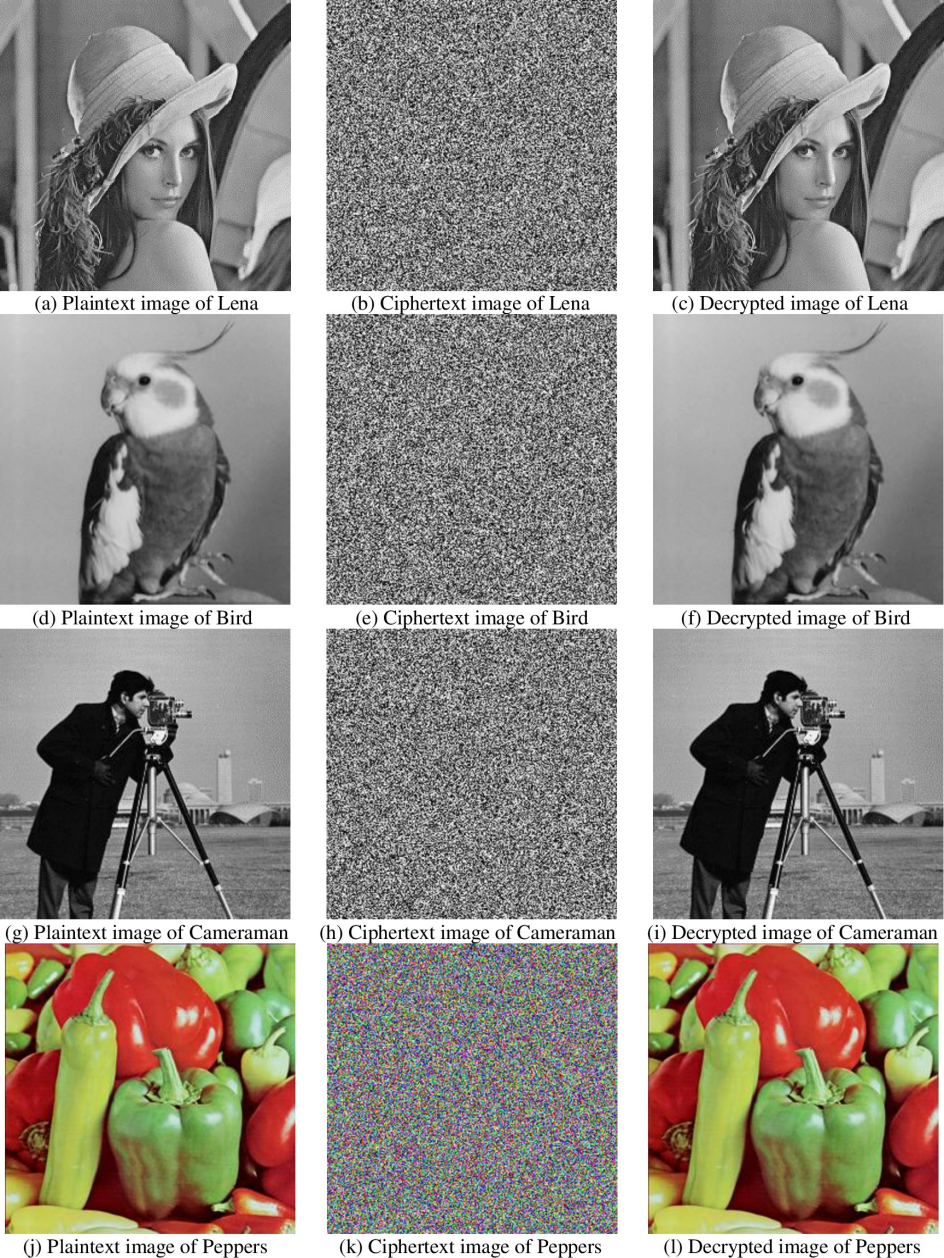
Plaintext, ciphertext and decrypted image of Lena, bird, cameraman, peppers.

This article selects an image of size 256×256 for encryption. Set *x* = 0.6,*μ* = 1.46,*x*_1_ = 0.1,*x*_2_ = *x*_3_ = *x*_4_ = *x*_5_ = 0.2, *q*_1_ = *q*_2_ = *q*_3_ = *q*_4_ = *q*_5_ = 0.98, Fig 5 shows the results of the encryption and decryption experiments on the images Lena, Bird, Cameraman and the color image Peppers.

### 4.2 Security analysis

#### 4.2.1 Violent attack

The key in this paper considers that the non-integer key has the precision of 10^−14^, so its key space should be 2^128^ or more, and the theoretical non-violent cracking has been achieved [20].

#### 4.2.2 Key sensitivity analysis

We make a minor change to one key, and the other keys remain unchanged to decrypt the image. The changed values are as follows:

$$D_{1}:x=0.6+10^{-14},\mu_{1}=1.46,x_{1}=0.1,q_{1}=0.98,$$

$$D_{2}:x=0.6,\mu_{1}=1.46+10^{-14},x_{1}=0.1,q_{1}=0.98,$$

$$D_{3}:x=0.6,\mu_{1}=1.46,x_{1}=0.1+10^{-14},q_{1}=0.98,$$

$$D_{4}:x=0.6,\mu_{1}=1.46,x_{1}=0.1,q_{1}=0.98+10^{-14},$$

**Fig 6:**
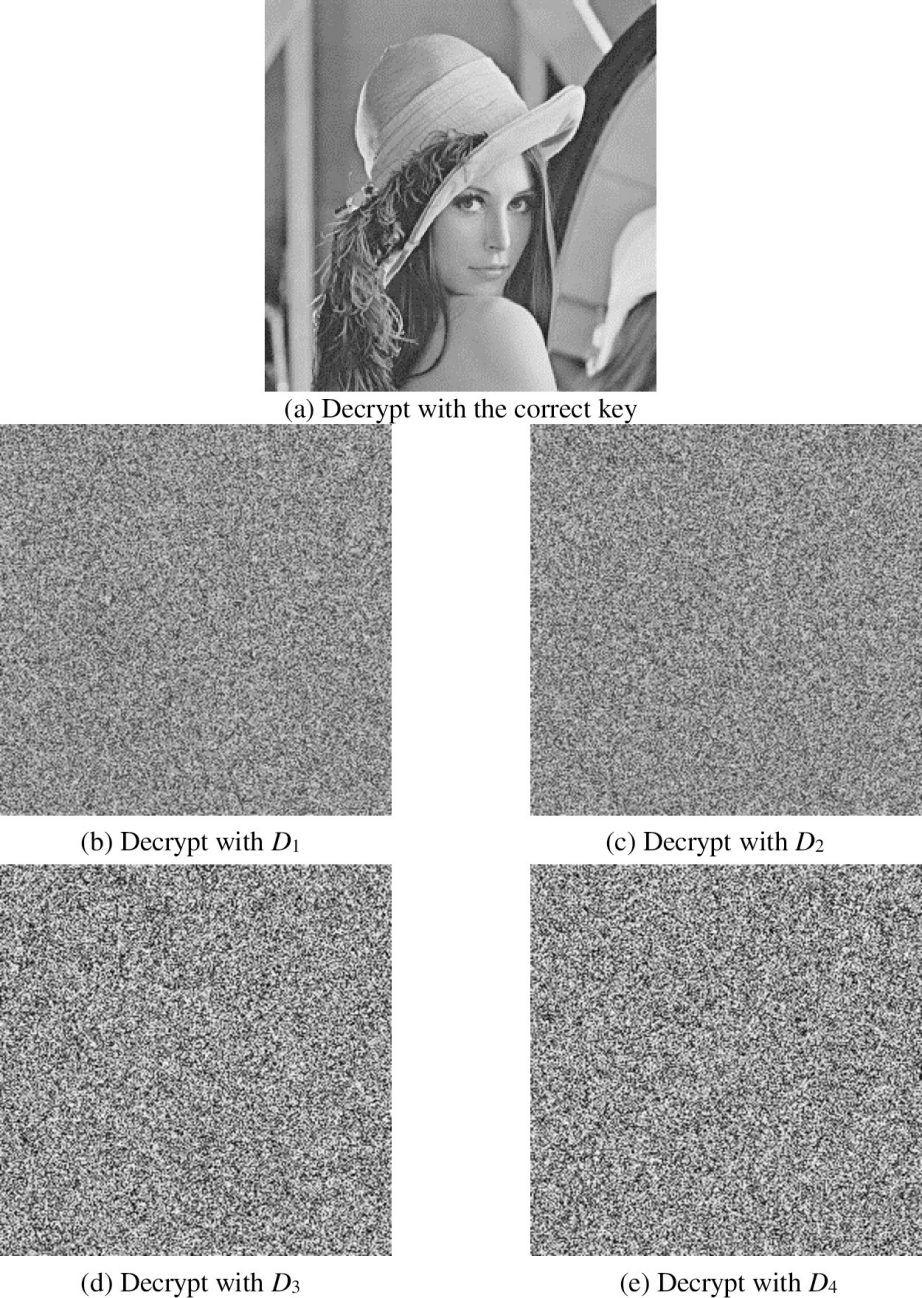
Decryption with different keys.

Fig 6 shows the decryption result. It can be seen that when the key is changed in the smallest order, the original image cannot be decrypted, so the algorithm is sensitive to the key.

#### 4.2.3 Key statistics analysis

Statistical analysis after image encryption is very important. A good image encryption algorithm should be able to resist any kind of statistical attack. Among them, the histogram analysis of images and the correlation of adjacent pixels are two very important statistical characteristic indicators in image encryption algorithms.

1 Histogram analysis

**Fig 7:**
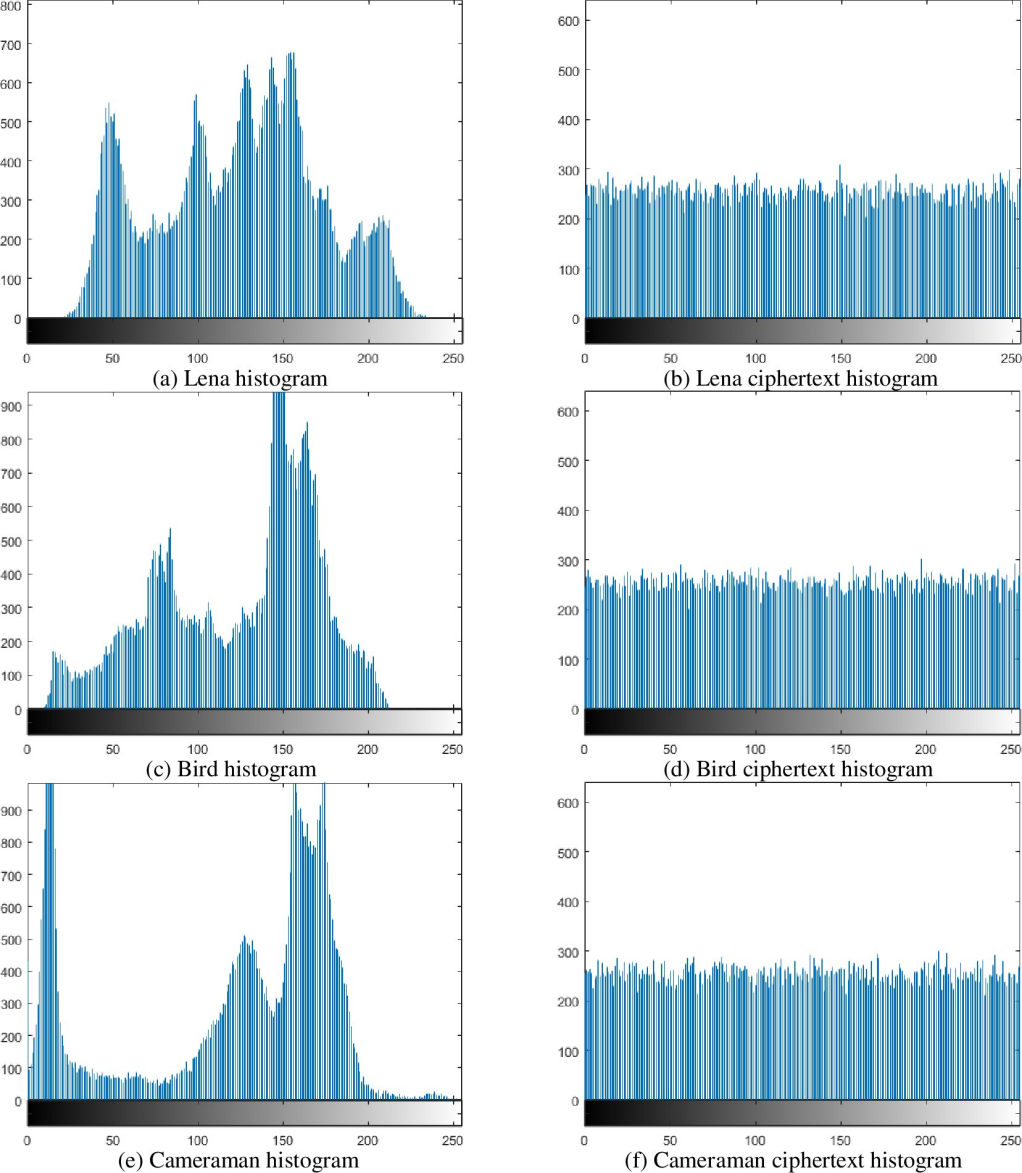
Plaintext and ciphertext histogram.

A histogram is a function graph of the number of pixels of a statistical image having the same attribute value. Generally, the more uniform the pixel values of the ciphertext image processed by the encryption algorithm are, the more uniform the histogram looks [21]. As can be seen from the results of Fig 7, the pixel value distribution of the ciphertext image is obviously more uniform.

2 Adjacent pixel correlation

In the plaintext image, adjacent pixels tend to have strong correlation. In order to avoid others using statistical information attacks, the correlation between adjacent pixels of the ciphertext image must be reduced [22]. The formula for calculating the pixel correlation is

$$r_{xy}=\frac{\mathrm{cov}(x,y)}{\sqrt{D(x)}\sqrt{D(y)}},$$ (17)

where

$$\left\{ \begin{array}{l} \mathrm{cov}(x,y)=\frac{1}{N}\sum_{i=1}^{N}\left(x_{i}-E(x))(y_{i}-E(y)\right)\\ D(x)=\frac{1}{N}\sum_{i+1}^{N}{\left(x_{i}-E(x)\right)}^{2}\\ E(x)=\frac{1}{N}\sum_{i=1}^{N}x_{i} \end{array}\right..$$ (18)

**Fig 8:**
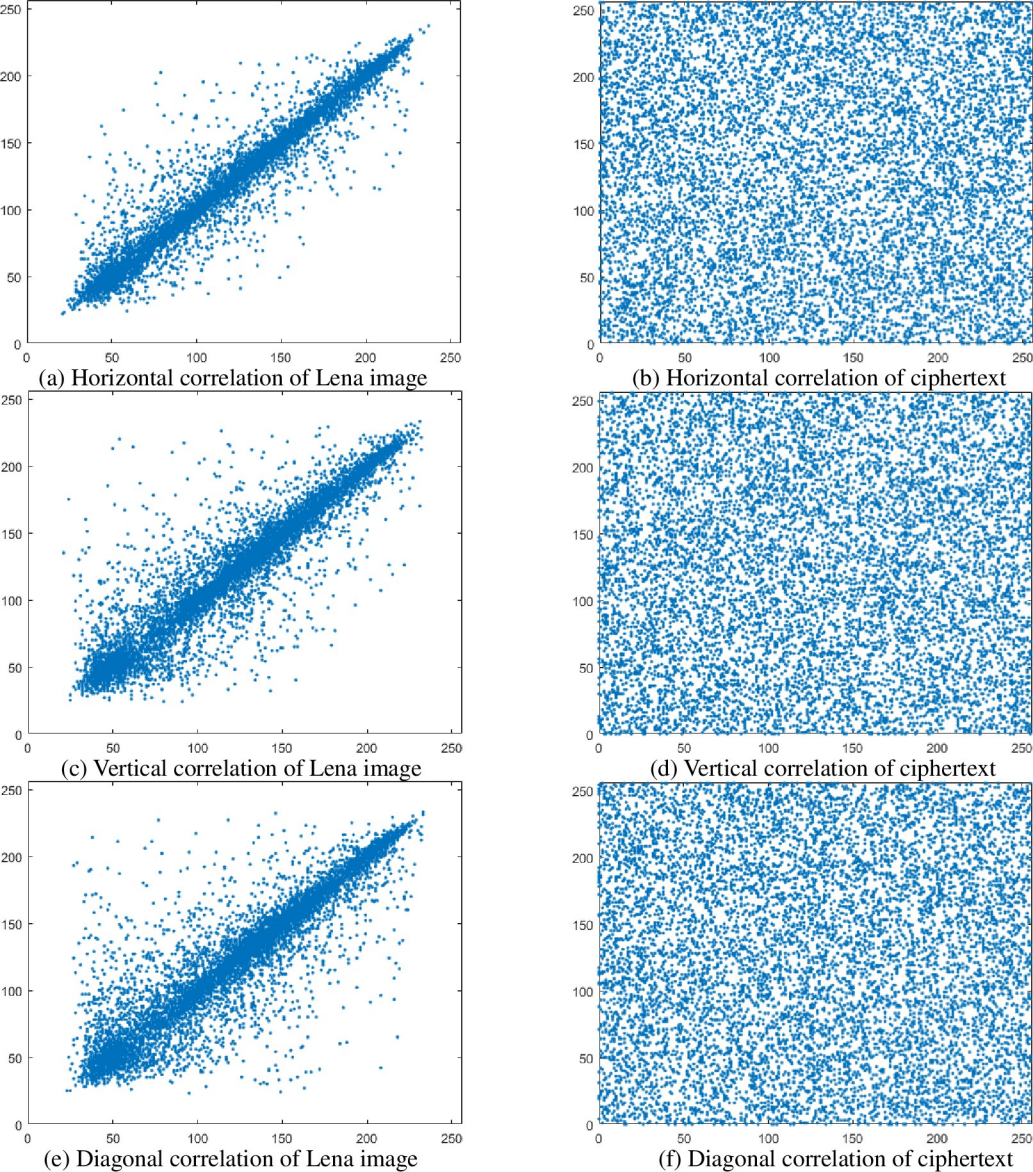
Correlation comparison of plaintext ciphertext.

In this paper, the horizontal, vertical and oblique correlations of plaintext and ciphertext are statistically calculated using Eqs (17) and (18). The statistical results are shown in Fig 8. Among them, the Ref. [23] is an algorithm using skewed Tent map and 6th-order CNN, and the literature [24] is an algorithm that uses two hyperchaotic systems for encryption. The Ref. [24, 26] is the current excellent experimental result. The subsequent comparison process goes into the information for the Lena image, and will not be described again. It can be seen from Table 1 that after the encryption of this algorithm, the correlation of the ciphertext is close to 0, and compared with other algorithms, this algorithm has a greater advantage, so it can resist statistical attacks.

**Table 1:** Correlation between plain image and adjacent pixels of ciphertext image.

Image	Horizontal	Vertical	Diagonal
Lena	0.8951	0.9650	0.9203
Ciphertext of Lena	-0.0002	0.0011	0.0965
Bird	0.9826	0.9810	0.9889
Ciphertext of Bird	-0.0007	0.0024	0.0013
Ref. [23]	-0.0168	0.0445	-0.0022
Ref. [24]	-0.0062	0.0052	0.0043
Ref. [25]	-0.0318	0.0965	0.0362
Ref. [26]	0.0051	-0.0093	-0.0205

3 Information entropy analysis

Information entropy is a way to evaluate the encrypted image. The closer the information entropy value is to 8, the more disordered the encrypted image [26]. The calculation formula of information entropy is as follows:

$$H(s)=\sum_{i=0}^{2^{L}-1}p(s_{i})\log_{2}\frac{1}{p(s_{i})}$$ (19)

where *p*(*s*_*i*_) is the probability of occurrence of *s*_*i*_.

Table 2 shows the comparison between this algorithm and other algorithms. It can be seen that the encrypted image is more disordered and can resist statistical attacks.

**Table 2:** Information entropy comparison result.

Image	Information entropy
Lena	7.2775
Ciphertext of Lena	7.9989
Bird	7.2577
Ciphertext of Bird	7.9896
Ref. [23]	7.9744
Ref. [24]	7.9993
Ref. [25]	7.9851
Ref. [26]	7.9991

#### 4.2.4 Differential attack analysis

Differential attacks are a choice of plaintext attacks. That is, by making a slight change to the plaintext, the ciphertext and the modified ciphertext before the encryption algorithm are modified, and the data is analyzed to obtain the key. Therefore, a good image encryption system should be able to make small changes in plaintext can also cause huge changes in ciphertext to be able to withstand differential attacks [27]. The Number Pixels Change Rate (NPCR) and the Unified Average Changing Intensity (UACI) are respectively

$$NPCR=\frac{\sum_{i,j}D(i,j)}{W\times H}\times 100\%,$$ (20)

and

$$UACI=\frac{1}{W\times H}[\sum_{i,j}\frac{\left|C_{1}(i,j)-C_{2}(i,j)\right|}{255}]\times 100\%.$$ (21)

Where *W* and *H* represents the width and height of the image. *C*_1_ and *C*_2_ are the two ciphertext images after the original plaintext image changes by one pixel value. If *C*_1_≠*C*_2_, then the corresponding *D*(*i*,*j*) = 1, otherwise *D*(*i*,*j*) = 0. The larger the value of NPCR, the more sensitive the encryption algorithm is to the changes in the original plaintext image; the larger the value of UACI, the greater the average change intensity [24]. Under ideal conditions, the closer the NPCR is to 99.6049%, the better the UACI is closer to 33.4635%. Table 3 is a comparison between this algorithm and other algorithms. It can be seen from the table that although it is not very close to the ideal value, it can better resist differential attacks.

**Table 3:** NPCR and UACI comparison results.

Image	NPCR	UACI
Ciphertext of Lena	99.6302	33.4521
Ciphertext of Bird	99.6218	33.4375
Ref. [22]	99.5643	35.4560
Ref. [23]	99.6002	33.3635
Ref. [24]	99.6140	33.4828
Ref. [25]	99.6239	33.6623

#### 4.2.5 Cutting and noise attacks

**Fig 9:**
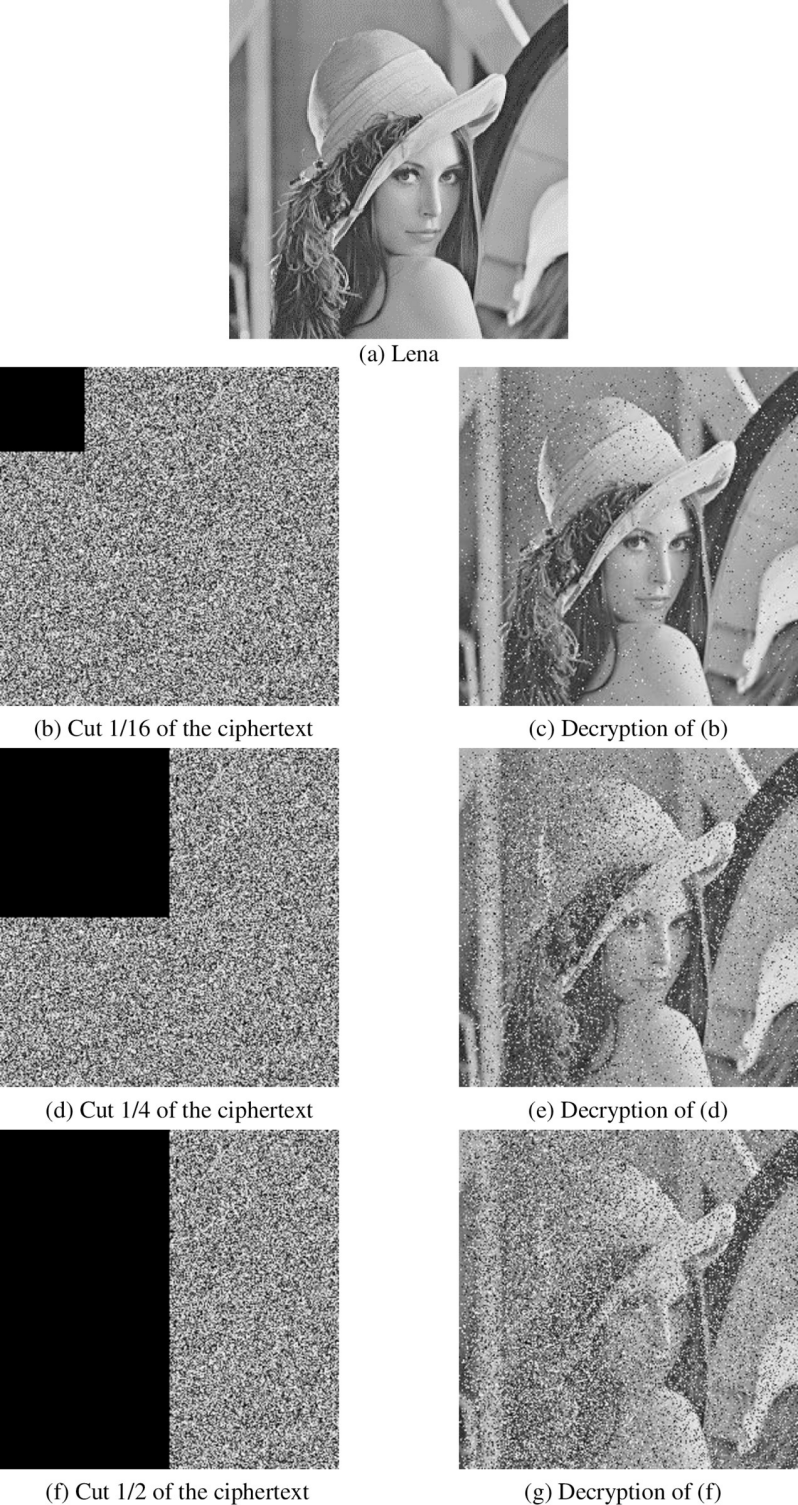
Cutting attack resistance.

**Fig 10:**
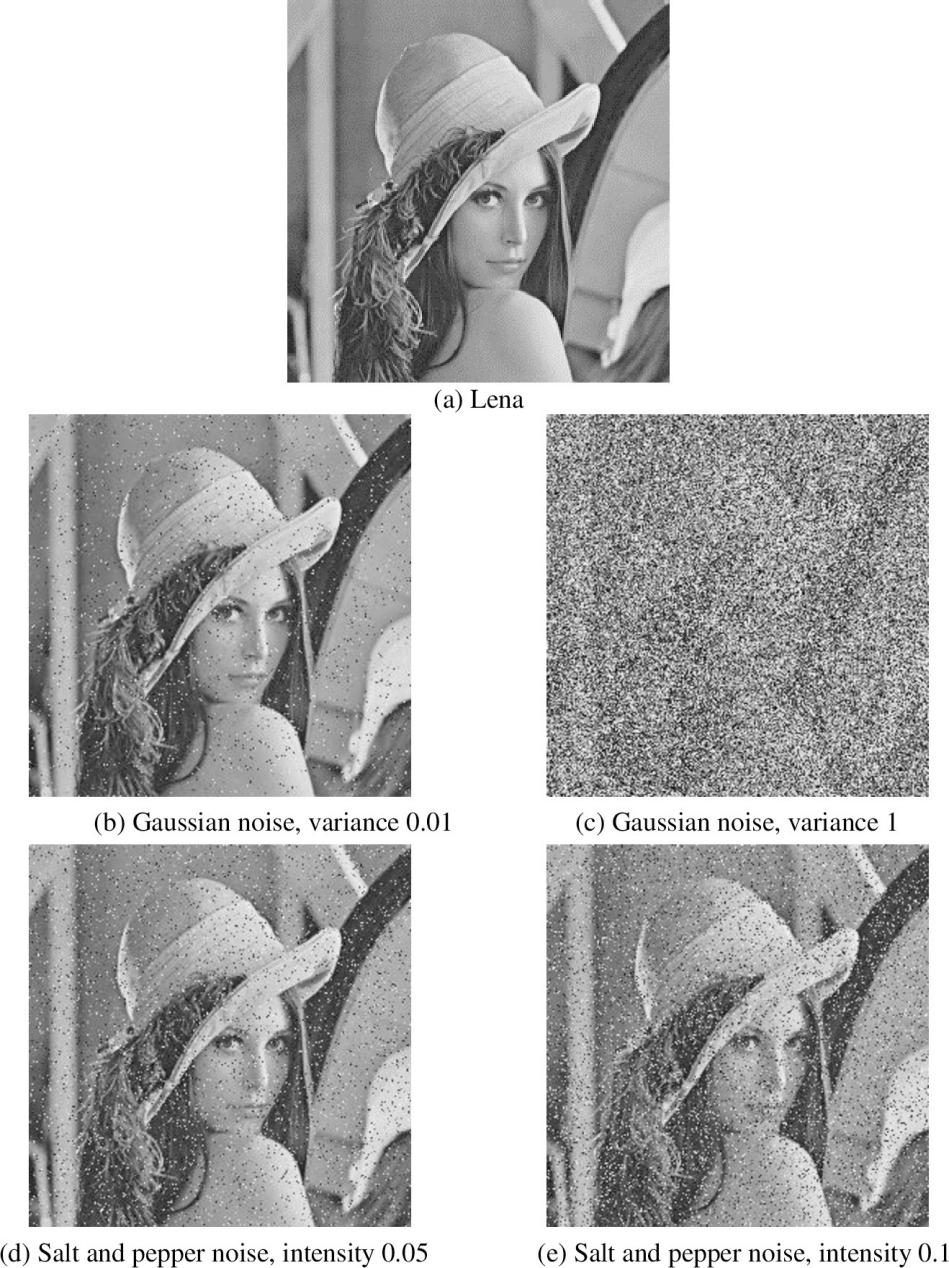
Noise attack resistance.

In the process of communication, if signal hijacking is encountered, the transmitted ciphertext may be tampered with. Therefore, in order to prevent malicious hijacking and modify ciphertext, ciphertext should have good performance against scratch attacks. In this paper, the ciphertext is clipped at different positions of 1/16, 1/4, 1/2, and decrypted using the clipped ciphertext. There are not only cutting attacks but also noise attacks. In order to detect the ability to resist noise attacks, this paper uses different strength Gauss noise, salt and pepper noise to attack, as shown in Figs 9 and 10. It can be seen that the encryption algorithm in this paper can resist cutting attacks and noise attacks.

#### 4.2.6 Deviation from uniform histogram

In an ideal encryption model, the encrypted image should have a uniform histogram distribution to hide pixel related information. This means that the encryption algorithm changes the cryptographic pixel values to make the probability of each cryptographic pixel completely uniform. Ref. [28] gives a method for estimating the quality of encryption, uniform histogram deviation (*D*_*H*_), it is given by Eq (22),

$$D_{H}=\frac{\sum_{C_{i}}^{255}\left|H_{C_{i}}-H_{C}\right|}{M\times N},$$ (22)

where *M*×*N* is the size of the image, $H_{C_{i}}$ Is the statistic of the ciphertext image at the pixel value of *i*, *H*_*C*_ is a standard uniform histogram, the statistics of each pixel are *M*×*N*/256. It can be seen that when *D*_*H*_ is smaller, it proves that the more uniform the pixel distribution of the ciphertext, the better the encryption effect. We chose some similar chaotic neural networks, as well as some excellent results in some areas for comparison. At the same time, for comparison, this paper also selected the same encrypted images as the comparison references. As shown in Table 4, the *D*_*H*_ value of this algorithm is significantly smaller than other algorithms.

**Table 4:** Standard histogram deviation comparison result.

Image	Proposed algorithm	Ref. [24]	Ref. [29]	Ref. [30]	Ref. [31]	Ref. [32]
Peppers	0.0531	0.0492	0.0938	0.0979	0.0917	0.0977
Airplane	0.0547	0.0518	0.0969	0.0995	0.0983	0.0943
Boat	0.0503	0.0524	0.0902	0.0995	0.0958	0.0985

#### 4.2.7 Time analysis

In addition to the security analysis of the algorithm, in practice, the encryption algorithm has a fast encryption speed. In order to test the encryption speed of this algorithm, we tested the encryption speed of three images of size 256×256, run the program 100 times, and obtained the average operation time. As shown in Table 5, we can see that the encryption time of different images is about 1 second, which is more advantageous in time than the literature [8], [10]. Table 6 shows that for the Lena graph, this algorithm is compared with other algorithms. The results show that this algorithm is good in time efficiency.

**Table 5:** Execution time with the same operating environment (unit: s).

Image	Proposed algorithm	Ref. [8]	Ref. [10]
Lena	0.981488	4.230902	1.682497
Cameraman	1.008242	4.330022	1.784329
Peppers	1.007021	4.482100	1.747320

**Table 6:** Encryption time and comparisons.

Algorithm	Encryption Time (sec)
Proposed algorithm	0.981488
Ref. [8]	4.230902
Ref. [10]	1.682497
Ref. [20]	1.676400
Ref. [33]	1.205000

## 5 Conclusion

In this paper, two systems are used to encrypt the image. In order to generate a chaotic sequence when using a staged compound chaotic map, the image is randomly scrambled by Fisher-Yates. Using a fractional 5D cellular neural network can increase the complexity of the system, and then serialize the matrix generated by it, and diffuse the image information through XOR processing with the key and the ciphertext to obtain the ciphertext image. Key space analysis, statistical information analysis, differential attack analysis, cropping attack, noise attack, etc., prove the superiority of the algorithm and good resistance to attacks.
